# Validation of the Short Food Literacy Questionnaire in the Representative Sample of Polish Internet Users

**DOI:** 10.3390/ijerph19159710

**Published:** 2022-08-06

**Authors:** Urszula Zwierczyk, Mateusz Kobryn, Mariusz Duplaga

**Affiliations:** Department of Health Promotion and E-Health, Institute of Public Health, Faculty of Health Sciences, Jagiellonian University Medical College, 31-066 Kraków, Poland

**Keywords:** food literacy, health literacy, nutritional habits, exploratory factor analysis, confirmatory factor analysis, validation, short food literacy questionnaire

## Abstract

Analogous to health literacy, food literacy can be defined as a set of cognitive and social skills associated with the ability to acquire and understand information about food and nutrition to make appropriate nutritional decisions. In the literature, several terms such as food, nutrition, or nutritional literacy are used in parallel, differing in some aspects of their meaning. Food literacy is an important measure of the effectiveness of nutritional education interventions and appropriate instruments for its measurement should be available in every society. The aim of this study was the assessment of the validity and testing of a proposed model of the Short Food Literacy Questionnaire (SFLQ) culturally adapted into Polish. The analysis was performed on data from an online survey in a representative sample of 1286 adult internet users. Exploratory (EFA) and confirmatory factor (CFA) analyses were performed on two different subsets obtained through random splitting of the initial dataset. The Polish version of the SFLQ had good internal consistency (Cronbach’s α 0.841; Guttman split-half coefficient was 0.812). The EFA revealed that the tool had a three-factor latent structure. The distinguished dimensions were ‘information accessing’, ‘knowledge’, and ‘information appraisal’. The subscales also showed acceptable internal consistency based on the values of the Cronbach’s α coefficients (ranging from 0.768 to 0.845). The CFA confirmed a good fit of the three-factor model with at least five indexes achieving acceptable levels (CFI = 0.972, GFI = 0.963, AGFI = 0.940, NFI = 0.959, and RMSEA = 0.059). The validation of the Polish version of the SFLQ revealed, contrary to earlier reports, not a single but a three-factor structure of the instrument. The SFLQ will be an important tool for the assessment of the effectiveness of educational interventions and population studies analyzing the determinants of food literacy in Poland.

## 1. Introduction

Health literacy (HL) is defined as cognitive and social skills that define an individual’s motivation and ability to obtain, process, understand, and use information in ways that lead to improved and maintained health [1]. An integrated model of HL developed by the European Health Literacy Project (HLS-EU) assumes that there are four main skills related to handling health information: accessing, understanding, appraising, and applying [2]. Furthermore, HL can be relevant to health information in three domains: health promotion, diseases prevention, and healthcare. HL depends on many factors, including sociodemographic variables, cultural background, and earlier contacts with the healthcare system [2]. A higher level of HL is associated with making appropriate health-related decisions and improved well-being [3]. Societies with higher HL are able to promote more effective health policies and diminish health inequities. HL is an important concept that makes it possible to anticipate a patient’s adherence to preventive and therapeutic interventions and even prognosis. It has been reported that limited HL is associated with inadequate adherence to recommended cancer screening interventions, the inability to decide about therapeutic options, and a lower quality of life after cancer diagnosis [4]. Another study confirmed that quality of life also depends on the level of HL in patients with acute coronary syndrome [5]. An international study carried out within the HLS-EU project confirmed that persons with lower HL more often declare unsatisfactory health status, have a higher prevalence of chronic diseases, and more often utilize healthcare resources [6].

It is clear that HL is a complex concept associated with individual behaviors and lifestyle as well as the way we interact with the healthcare system. Nutrition is one of the key elements of a healthy lifestyle. Adequate knowledge about food and nutrition and the ability to shape beneficial dietary behaviors and maintain normal body weight are indicated as important aspects of HL that add to wellbeing and prevent chronic diseases. The appreciation of the nutritional aspects of health promotion results in the formulation of the HL-related concepts called nutrition, nutritional, or food literacy [7,8]. The differences between these terms are not always fully clear, and available definitions overlap. The definition of nutrition literacy was coined, by analogy to HL, as the ability to acquire and understand information about food and nutrition to make appropriate nutritional decisions [9]. Some authors have proposed that this definition should be widened to include practical skills of using knowledge and communicating with professionals [10]. The capacity to appraise the quality of nutrition information is another aspect usually included in the definition of nutrition literacy [10]. As for food literacy (FL), it is defined as a set of skills related to acquiring and processing information about food, nutrition, and its application in everyday life, and the ability to prepare healthy meals and understand the impact of food on health, the environment, and the economy [11]. Sumner emphasized that food literacy should address the entire food production chain: from where and how food is produced, who gains and who loses from purchasing it, who has access to food and who does not, and, finally, how it is disposed of [12]. Furthermore, Sumner’s perspective also includes cultural, environmental, social, and economic contexts of food and its importance within national policies.

By analogy to Nutbeam’s taxonomy of HL [13], three categories of food literacy (FL) can be distinguished: functional, interactive, and critical. The first category refers to understanding and using information about food and nutrition, e.g., for cooking, shopping, and preparing a balanced menu. Interactive FL refers to the skills related to communicating and exchanging nutrition information with family and friends. According to Perry et al., it should also encompass the ability to read and understand food labels and, based on them, select products with high nutritional value [14]. Finally, critical FL is the ability to assess the reliability of food information communicated by other people, nutrition professionals, or commercial entities [7,15]. Other authors have proposed that assessing the long-term impact of eating behaviors on an individual’s health should be included in critical FL [7]. 

In a systematic review published in 2018, Yuen et al. described 13 tools for the assessment of nutrition and food literacy [8]. The most popular scales include the Nutrition Literacy Scale [16], the Nutrition Literacy Assessment Instrument [17], the Newest Vital Sign [18], the Electronic-Nutrition Literacy Tool [19], and the Short Food Literacy Questionnaire (SFLQ) [20]. None of these tools have been adapted into Polish so far. The authors of this paper are reporting the results of the cultural adaptation and validation of the SFLQ, which was originally developed by Krause et al. [20] for a survey performed among the Swiss population. This tool was selected for adaptation due to its brevity and the feasibility of applying it within broader studies focusing on health literacy, behaviors and lifestyles, and the preparedness to respond to health-related challenges.

The intention behind the development of the SFLQ was to enable the measurement of skills corresponding with functional, interactive, and critical elements of FL [20]. Krause et al. underlined that their tool focused on the individual skills and abilities needed for healthy food choices. The questionnaire consists of 12 items asking about the ability to find information about healthy nutrition and understand the nutritional information provided by various sources, the knowledge of national guidelines on nutrition, the ability to compose a balanced meal and help closest friends and family concerning nutritional issues, the ability to assess the reliability of information and commercials, and, finally, the ability to predict the relevance of specific foods to a healthy diet or to predict the prolonged impact of dietary habits. The original version of the SFLQ was developed in German for a study among the Swiss population [20]. The authors reported that this instrument had a one-factor latent structure and adequate construct validity.

The SFLQ was adapted to Turkish by Durmus et al. who also reported that the tool had a one-factor latent structure and good internal consistency [21]. They found a positive moderate correlation between the FL score based on SFLQ and the HL score calculated on the Turkey Health Literacy Scale-32 (TSOY-32) among the sample of adult respondents. The next study in a group of university students from Turkey revealed that the SFLQ score is significantly positively associated with general health perception and the habit of reading food labels [22]. The Italian version of the SFLQ was used by Trieste et al. in the study conducted among Italian consumers [23]. Their analysis showed that the SFLQ score was significantly associated with purchasing behaviors, gender, and the place of residence. Recently, Itzkovitz et al. observed a significant relationship between the SFLQ score and higher cooking skills and confidence in preparing healthy meals among young Canadian adults living with type 1 diabetes [24].

The main aim of our study was the validation with exploratory factor analysis (EFA) of the Polish adapted SFLQ and the assessment of the proposed model with confirmatory factor analysis (CFA) on two subsets of data originating from the survey in the representative sample of adult internet users. 

## 2. Materials and Methods

The Polish version of the SFLQ instrument was prepared following the World Health Organization guidelines for cultural adaptation [25]. The data for the analysis described in this paper were obtained from an online survey performed in a representative sample of 1286 adult internet users. The initial dataset was randomly split into two subsets. The first one was used for the EFA and the second for CFA. In the first step, the internal consistency of the questionnaire was assessed. Before the EFA, the sample size in relation to the number of items was analyzed and the factorability of the data was assessed. Multicollinearity was excluded after considering the correlation matrix. The latent structure of the SFLQ instrument adapted to Polish was assessed with EFA based on the maximum likelihood method. For the assessment of the fit of the model, several indexes generated with CFA were applied.

### 2.1. Survey

The data used for the validation of the SFLQ were obtained from a computer-assisted web-based interviewing (CAWI) survey given to 1286 adult internet users in December 2021. Respondents were recruited by a third party, the Ogólnopolski Panel Badawczy Company, which maintains an internet panel (Ariadna panel) [26]. The company was selected as a result of the tender procedure obligatory for public organizations in Poland. The sample was established as a result of a stratified proportional sampling from a certified internet panel to reflect the structure of the population of Polish internet users concerning age, education, place of residence, and Nomenclature of Territorial Units for Statistics (NUTS) 1 region.

The study reported in the paper was conducted after receiving the consent of the Bioethical Committee of Jagiellonian University in Krakow (Decision No 1072.6120.99.2020 from 23 April 2020, with amendments).

### 2.2. Questionnaire

The questionnaire used in the survey consisted of 86 items. It included the 16-item European Health Literacy Survey questionnaire (HLS-EU-Q16) [6], the 12-item Short Food Literacy Questionnaire (SFLQ) [20], the items from the Eating Motivation Scale (EATMOT) [27] related to the environmental and marketing aspects of decisions related to food selection, a set of items asking about nutritional behaviors and other health-related behaviors, and items asking about the sociodemographic and economic status of the respondents. 

The basic version of the European Health Literacy Survey Questionnaire (HLS-EU-Q) consists of 47 items [28]. In this study, the 16-item version of the questionnaire (HLS-EU-Q16) was used. The respondents are able to provide responses to questionnaire items on a scale from very difficult to very easy. The response options, ‘very difficult’ and ‘fairly difficult,’ are assigned a value of 0, and the response options ‘fairly easy’ and ‘very easy’ are assigned a value of 1. The response ‘difficult to say/not applicable’ is treated as a missing value. The total score based on the HLS-EU-Q16 is calculated as the sum of values assigned to the individual items if the number of missing values is not greater than 80%.

The EATMOT scale was developed by Guiné et al. for the assessment of the motivations responsible for eating choices [27]. It was validated in an international survey performed within a European project. In this study, only items asking about the role of environmental and political motivations and marketing and commercial motivations were included. Responses are provided based on a 5-point ordinal Likert scale from strongly disagree to strongly agree with the neutral response in the middle. The items of this scale were not used in the analysis presented here. 

### 2.3. The Polish Version of the SFLQ

The Polish version of the SFLQ (PL-SFLQ) was developed in line with the modified guidelines for transcultural adaptation from the WHO [25]. Permission to use the tool was received from Dr Corinna Krause, representing the authors who developed and validated the Swiss version of the tool [20]. The forward translation of the SFLQ was prepared by two native-Polish speaking professionals with advanced German language skills. The translators were advised that they should aim at the conceptual equivalent and not a word-for-word translation and retain the concepts of the original version while using expressions appropriate for Polish cultural contexts. Furthermore, the emphasis was put on clearly and concisely formulating the items and avoiding long sentences. Finally, the language of the translation was to be appropriate for the most common and not professional audiences. The use of jargon was discouraged. 

The translated versions were discussed and the final version was adapted by an expert five-person panel, including three researchers from the Department of Health Promotion and e-Health of the Jagiellonian University Medical College with interdisciplinary backgrounds in public health, nutrition, and sociology, and two collaborating members with a linguistic background. Phrasing of items was discussed and accepted by consensus. It was agreed that in item 3, both ‘Health Nutrition Pyramid’ and ‘Healthy Nutrition Plate’ would be referred to, as the latter concept had been included in national Polish recommendations only recently before starting the adaptation process [29]. 

The consensus version of the questionnaire was back-translated by an independent translator whose mother tongue was German who had no knowledge of the original questionnaire. The translator provided back-translation of the whole questionnaire with special attention to key terms for the theme of the tool and those that could be prone to difficulties in forward translation due to transcultural differences. Overall, the comparison of the original and back-translated versions of the questionnaire did not reveal major discrepancies and the version of the tool accepted by the expert panel was retained for further activities. 

Pretesting of the questionnaire was conducted in a group of 12 respondents representing the general population but not anticipated to join the main survey. The pilot group consisted of 6 females and 6 males. The mean age (standard deviation) in the pilot group was 43.6 (12.3) years. The participants represented various levels of education: 50% were married; most of them were employed in the public or private sector. They were provided with paper questionnaires with space for their feedback after the SFLQ items had been answered on their thoughts and why they had chosen their answers. Furthermore, the respondents were asked to indicate the words or phrases not fully clear to them. After filling out the paper questionnaire and responding to the questions about responding to the SFLQ items, a team member (UZ) discussed their feedback and asked about their potential doubts. The use of the paper form as a first stage of the cognitive interview followed by a shorter face-to-face wrap-up meeting was dictated by the epidemiologic situation of the COVID-19 pandemic. The results of the pre-testing and the cognitive interviewing were discussed by the expert panel. A minor modification was introduced to the phrasing of item 12. The suggestions provided regarding items 6 and 7 by one of the respondents were deemed not fully appropriate in terms of grammar and were also assessed as leading to more complex and difficult-to-understand sentences. Overall, the understanding of the items included in the SFLQ was good and no terms were indicated as unclear.

Original German, Polish, and English versions of the items included in the SFLQ are provided in the Supplementary Material File: Table S2.

### 2.4. Statistical Analysis

Statistical analysis was performed with the IBM SPSS v.26 and IBM SPSS Amos 26 software (IBM Corp, Armonk, NY, USA). Descriptive statistics were provided for relevant variables: absolute and relative frequencies for categorical variables and means and standard deviations (SD) for continuous variables. 

The internal consistency of the Pl-SFLQ tool was assessed with Cronbach’s α coefficients. It was assumed that a value of Cronbach’s α coefficient between 0.7 and 0.9 indicates good and ≥0.9 indicates excellent internal consistency. As a test–retest analysis was not performed during the survey, the Guttman split-half coefficient was calculated. A Guttman split-half coefficient value of at least 0.80 was needed to confirm the internal consistency of the scale. The percentage of participants who scored 6 and 53 were used to assess floor and ceiling effects, respectively. For each item, item-to-total-score correlations were calculated.

The adequacy of the sample size in relation to the number of items included in the SFLQ was analyzed with the Kaiser–Meyer–Olkin test (an expected value >0.70 is suggested as good by Hutcheson and Sofroniu [30]). The factorability of the data was assessed with the Bartlett’s test of sphericity. The multicollinearity was analyzed based on the correlation matrix. It was recommended that once of a pair of items correlated above 0.8, they should be removed [31]. 

The validity of the scale was examined with an exploratory factor analysis (EFA). EFA factoring was applied to discover the underlying latent variables responsible for the variance of the measure. The extraction of factors was based on the maximum likelihood method. The analysis was performed on the subset of survey data received by its random splitting after applying the functionality available in the SPSS package. Dimension reduction is used to identify items with shared variance, so items with low communalities are usually removed. It was assumed that the communality score should be at least 0.2 [32]. 

The Kaiser criterion (eigenvalue of extract factor of at least 1.00) was applied for the extraction of factors. A scree plot was developed to display the factors that are to be retained. The principal factors were extracted after varimax orthogonal rotation. A value < 0.3 was assumed as a suppressing factor loading [31]. Scores >0.4 were considered stable [33]. It was also assumed that items should not cross-load significantly between factors (the ratio of loadings < 75%). It was expected that the extracted factors should have at least three items without cross-loading and with a sufficient loading score. Furthermore, it was also assumed that the retained factors should explain at least 50% of the total variance [34]. 

The construct validity of the tool was performed through hypothesis testing. The correlations of the food literacy score based on the SFLQ with health literacy and scores reflecting selected nutritional behaviors were determined. 

Confirmatory factor analysis (CFA) was performed on the second subset of data after random splitting of the initial dataset to verify the factor structure of the 11-item SFLQ, after the earlier exclusion of item 7. The overall fit of the hypothesized factorial model and estimation of the construct’s effects on the measured variables was then analyzed. The estimation method was maximum likelihood. The CFA was performed for the two models of the SFLQ, first including one factor as reported by Krause et al. for the original Swiss version of the questionnaire [20] and by Durmus et al. for the Turkish adaptation of the scale [21]. The second model assumed the three-factor structure suggested by the EFA analysis of the Polish-adapted version. 

Several model fit coefficients were applied to assess the goodness-of-fit of the model in the dataset: chi2 statistics, the chi2-to-degrees-of-freedom ratio (CDFR), the-goodness of-fit index (GFI), the GFI-adjusted degrees of freedom (AGFI), the normed fit index (NFI), Bentler’s comparative fit index (CFI), and the root-mean-square error of approximation (RMSEA). Based on the available literature, the expected values assumed for these indexes were as follows: >0.05 and <2.0 for CDRF, ≥0.85 for GFI, ≥0.80 for AGFI, ≥0.90 for NFI, >0.95 for CFI, and, for the RMSEA value, <0.05 as good and 0.05–0.08 as acceptable fit [35,36,37]. The initial assumption was that at least three adequacy indexes with values surpassing the expected reference levels should be obtained to confirm the goodness-of-fit of the data to factor structure [38]. 

## 3. Results

### 3.1. Characteristics of the Study Sample

The characteristics of the survey sample and both subsets obtained after random splitting of the initial sample are shown in Table 1. The mean age (standard deviation, SD) of respondents in the whole sample was 36.33: in subset 1–36.00 (10.21) years; in subset 2–36.68 (10.19) years. 

### 3.2. Internal Consistency

No significant floor or ceiling effects were observed in subset 1 (floor effect 0.3%, ceiling effect 0.2%). Cronbach’s alpha coefficient was 0.841, and the Guttman split-half coefficient was 0.812, supporting the internal consistency of the scale. As for the multicollinearity assessment, none of the bivariate correlations between items surpassed the value of 0.8. For all but item 7, bivariate correlations were higher than 0.275 and most of them were moderate or strong (Table 2). In the case of item 7, only one correlation was as high as a value of 0.312, and others were much lower (Table 2). The correlation of individual items to the total score ranged from 0.23 for item 7 to 0.68 for item 8. The values of Cronbach’s alpha after removing individual items were lower for all items apart from item 7 (Supplementary Material File S1 Table S1).

### 3.3. Exploratory Factor Analysis

Bartlett’s test of sphericity confirmed the factorability of the correlation matrix (chi2 = 3103.56. *p* < 0.001). The adequacy of the sample size was confirmed by the result of the Kaiser–Meyer–Olkin test (0.884). As the communality score for item 7 was only 0.14, it was removed from the scale. The EFA based on the maximum likelihood method showed the model consisting of three factors to be valid for the sample (Table 3).

**Figure 1:**
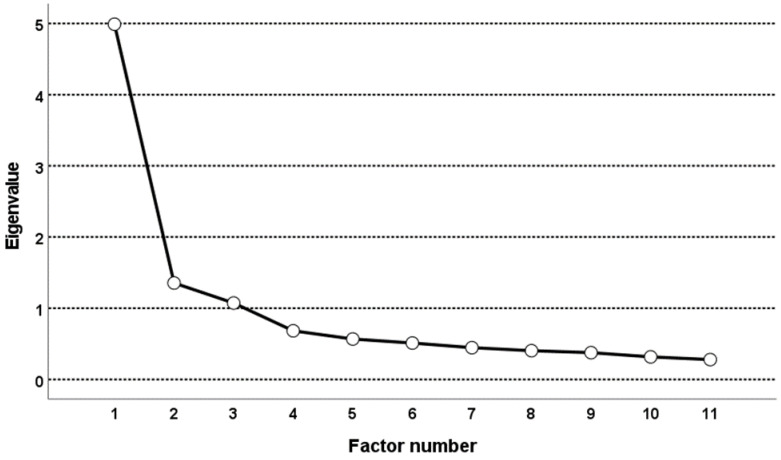
Scree plot.

The three-factor latent structure could also be seen on the scree plot (Figure 1). The initial eigenvalues for these three factors were 4.989, 1.355, and 1.073 (Table 3); they explained 67.43% of the total variance. After rotation, the eigenvalues of the extracted factors were 2.346, 1.943, and 1.894, respectively. The explained total variance was 56.02%.

After rotation, a minimum factor loading of >0.40 was achieved by all 11 items (Table 4). The lowest loading was found for item 6 (0.439); other loadings ranged from 0.589 to 0.818. The three factors obtained from the EFA were named as ‘Information accessing’ (factor 1) loaded with items 1, 2, 6, and 8; ‘Knowledge’ (factor 2) with items 3–5; ‘Information appraisal’ (factor 3) with items 9–12 (Table 4). The Cronbach’s α coefficient for the model consisting of 11 items, after exclusion of item 7, was 0.862; for factor 1—0.768; for factor 2—0.779; for factor 3—0.845.

Construct validity was tested based on the hypotheses validation. Descriptive statistics of the whole scale and three subscales and the results of the correlation analysis are shown in Table 5. It was assumed that the HL score derived from the HLS-EU-Q would be significantly correlated with the SFLQ score, and also, that the frequency of the consumption of certain types of food would be positively (e.g., fruit and vegetables) or negatively (e.g., red meat) correlated with FL, as measured with the SFLQ. The correlations of the FL score and self-assessed frequency of unfavorable eating habits, e.g., irregular meals or late suppers, were also calculated. Indeed, HL and FL scores were moderately correlated (Spearman’s ρ coefficient 0.46, *p* < 0.001) (Table 5). The dimension of SFLQ also showed positive correlation with HL; for ‘information accessing’, the correlation was 0.54; for ‘knowledge’—0.22; for ‘information appraisal’—0.42 (*p*-value for all correlation < 0.001). The frequencies of the consumption of fruit and vegetables, fish, and wholemeal bread were weakly significantly positively correlated with the SFLQ score. The frequencies of the consumption of red meat and industrial sugar products showed no correlation with the SFLQ score. Furthermore, four unfavorable nutritional habits were significantly weakly negatively correlated with the SFLQ score.

### 3.4. Confirmatory Factory Analysis

**Figure 2:**
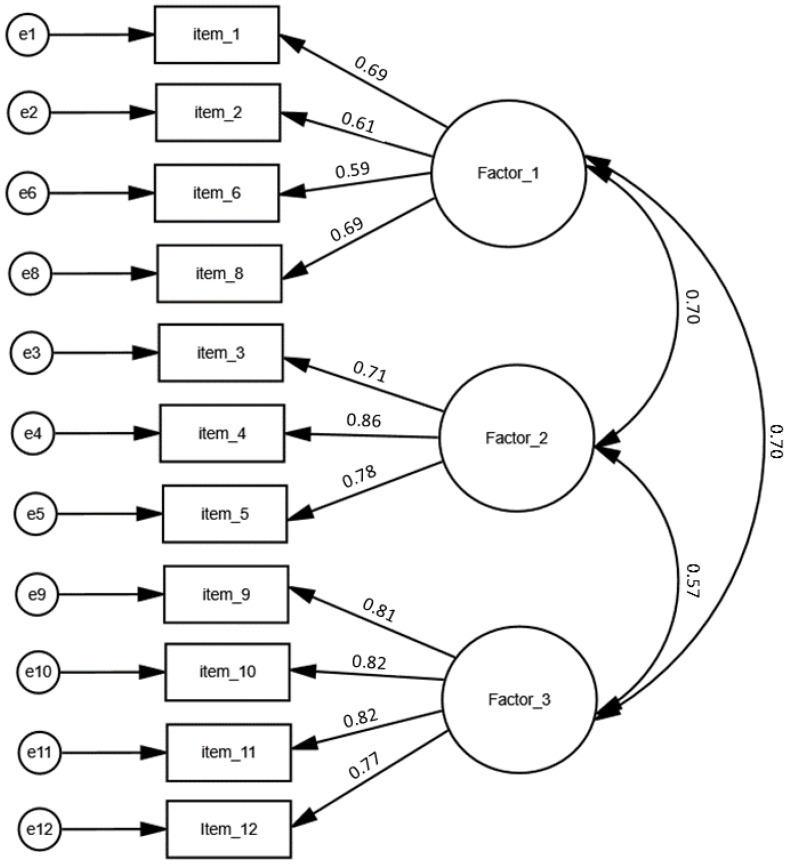
The CFA measurement model for the Polish version of the Short Food Literacy Questionnaire.

The CFA measurement model for the Pl-SFLQ is shown in Figure 2. Fitting results for the three-factor model and the threshold levels are presented in Table 6. All indexes used in the analysis, apart from chi2-to-degrees-of-freedom ratio (CDFR), showed good or at least acceptable fitting (Table 6).

As both the authors of the original German version of the SFLQ [20] as well as those of the Turkish adaptation [21] reported that the tool has a one-factor structure, the CFA was also developed for the one-factor model. The analysis showed that the one-factor model is not fitted based on the applied criteria (Table 6).

## 4. Discussion

In this study, data from a survey on a representative sample of adult internet users were used to validate a culturally adapted Polish version of the SFLQ, which was introduced initially by Krause et al. [20]. An EFA was performed in order to analyze the factor structure of the tool and compare it with the models reported by other authors [20,21]. The bivariate correlation coefficients were moderate or strong for all items apart from item 7. Item 7 asked ‘In the past, how often were you able to help your family members or a friend if they had questions concerning nutritional issues?’ In the case of this item, bivariate correlations were low. The correlation matrix did not show any bivariate correlation surpassing the value 0.8, which had been assumed as a sign of multicollinearity [31]. The communalities, meaning the ratios of items’ unique variances to their shared variance, have been calculated [32]. The assessment of communality coefficients showed that a threshold of 0.2 had not been reached by item 7. Furthermore, the Cronbach’s α coefficient of the SFLQ after excluding item 7 was higher than the coefficient before exclusion, increasing from 0.841 to 0.862. Therefore, the EFA analysis was re-run after removing item 7 from the scale. 

Our analysis revealed a multidimensional structure of the SFLQ on the representative sample of the population of internet users in Poland. Based on the EFA, three dimensions were proposed: ‘Information accessing,’ ‘Knowledge,’ and ‘Information appraisal.’ The factor, ‘Information accessing,’ was loaded with initial items 1, 2, 6, and 8. These items were related to the ability to search for, understand, and select information about food and nutrition. Item 6, asking about the perceived difficulty of composing a balanced meal at home in a usual day, was loaded to this factor, but it was seemingly related to more complex skills than only accessing information. Based on the factorial structure, item 6 was loaded with the lowest loading of all the items (0.439). The dimension, ‘Knowledge,’ was loaded with items 3–5 and was associated with the acquaintance with national guidelines about health nutrition. Finally, the dimension, ‘Information appraisal,’ was loaded with items 9–12, which addressed the challenge of assessing reliability and the potential influence of food on health in various contexts. The internal consistency of the three factors remained on an acceptable level, at least 0.768. These findings are contradictory to the findings reported by Krause et al. for the original German version of the tool, as they had confirmed a one-dimensional structure [20]. Similarly, Durmus et al. also reported a unidimensional structure for the Turkish version of the SFLQ [21]. 

The hypotheses testing showed acceptable construct validity of the Polish version of the SFLQ. A health literacy score based on the HLS-EU-Q16 showed moderate correlation with the SFLQ score. Furthermore, the SFLQ score correlated positively with the frequencies of the consumption of fruit and vegetables, wholemeal bread, and fish and negatively with unfavorable nutritional habits, e.g., irregular meals or skipping breakfast. These correlations were weak but statistically significant. The validation of the original Swiss version of the SFLQ revealed that the resulting FL score was positively associated with the ordered categories of HL based on the German version of HLS-EU-Q16 [20]. The study conducted among Turkish adults showed that the score based on the SFLQ is moderately correlated with the TSOY-32 score and weakly correlated with the Newest Vital Sign test [21]. Other studies showed that the SFLQ score was significantly associated with purchasing behaviors among consumers [23] and with skills and confidence in preparing meals in young adults suffering from type 1 diabetes [24]. 

It is well known that EFA is a data-driven and CFA is a theory-driven approach. It is usually accepted that an EFA is appropriate for the development of a scale and CFA should be used when a measurement model has developed a theory hypothesizing about patterns of loadings. The use of one of these methods depends on the aim of the study and it is discouraged that both methods be applied on the same dataset [39]. We applied an EFA on the first data subset obtained after random splitting of the survey sample to explore the factor model of the SFLQ. Earlier studies had shown this scale to have a single-factor structure [20,21]. However, it happens quite frequently that scales examined on other samples or adapted to other languages present different latent structures. In a related area, the e-Health Literacy Scale (eHEALS) introduced by Norman and Skinner may be an example [40]. The authors of the scale reported eHEALS to have a one-dimensional structure [40], but other studies also suggested two- [41] or three-factor models [42]. In this study, the CFA was conducted on another data subset obtained after random splitting of the initial data from the CAWI survey. The CFA was carried out in order to determine how much of the covariance between items is covered by the hypothesized factor structure and to assess the goodness-of-fit of the proposed model [43]. 

We assessed the model fit of the CFA based on several indexes, including chi-square statistics, CDFR, CFI, GFI, AGFI, NFI, and RMSEA. The threshold values for these indexes were derived from the relevant literature. The CFA was developed for the three-factor model obtained via EFA of the sample of the Polish internet user population and the one-factor model as reported earlier but after exclusion of item 7. The CFA confirmed that the three-dimensional model is well fitted. All indexes apart from chi-square statistics and CDFR showed at least an acceptable fit of the model. It should be noted that the feasibility of chi-square statistics as a model fit index may be affected by large samples. According to Babyak and Green, in such samples, the *p*-value decreases even in the presence of a small misfit [44]. Alavi et al. suggested that chi-square statistics with large sample sizes will probably remain statistically significant even after correlating pairs of error terms with the largest indexes to achieve the model fit during the exploratory phase of a CFA [45]. It is also recommended that the CDFR be used instead of chi-square statistics. The expected threshold for the CDFR spans from as high as 5.0 to 2.0 [43]; in the Polish sample, it was 3.15, so between the most liberal and conservative expectations. Five other indexes used in the CFA reached at least an acceptable level, including the RMSEA at 0.059. 

### Limitations

The analysis reported in this paper is based on data from a survey performed among adult internet users in Poland. It may be perceived as a limitation as nonusers were excluded from the analysis as they could present different patterns of nutritional behaviors and preferences regarding food choices. It should be considered that the internet is an important source of information about diet and food. Furthermore, some internet users are followers of healthy lifestyle influencers’ websites. The profile of the use of information sources by nonusers may result in different eating habits. Their economic and educational status may also be significantly different when compared to internet users. 

Due to the survey design and the fact that the survey was undertaken by a third party, test–retest analysis of data coming from a repeated survey among part of the sample was not performed. This analysis is planned to be conducted in another study with the SFLQ. 

At this stage, no other tools for the assessment of food literacy are available in the Polish version. Therefore, the hypothesis testing was carried out with the use of a general health literacy instrument and with the analysis of the frequencies of the consumption of selected food types and eating habits.

## 5. Conclusions

The EFA showed that the culturally adapted Polish version of the SFLQ has a three-factor structure. A CFA performed on another subset of data obtained after random splitting of the initial data from the CAWI survey performed among adult internet users confirmed a good fit of the three-factor model. To the authors’ knowledge, this is the first tool enabling the assessment of food literacy available in Polish. The questionnaire has good construct validity and internal consistency. It may be used for versatile purposes in the assessment of food literacy among adult populations and the effectiveness of nutritional educational interventions. Further studies would be needed to confirm its validity for groups of respondents not using the internet.

## Figures and Tables

**Table 1:** Characteristics of the study sample and subsets used for the exploratory and confirmatory factor analysis.

Variable	Variable Categories	All Respondents (n = 1286)		Subset 1 (n = 628)		Subset 2 (n = 658)	
		%	n	%	n	%	n
Gender	Female	49.53	637	48.73	306	50.30	331
	Male	50.47	649	51.27	322	49.70	327
Place of residence	Rural	38.34	493	36.46	229	40.12	264
	urban below 20,000 inhabitants	13.30	171	12.42	78	14.13	93
	urban 20,000–100,000 inhabitants	19.60	252	20.06	126	19.15	126
	urban 100,000–200,000 inhabitants	8.24	106	8.12	51	8.36	55
	urban 200,000–500,000 inhabitants	9.33	120	9.55	60	9.12	60
	urban above 500,000 inhabitants	11.20	144	13.38	84	9.12	60
Education	lower than secondary	11.90	153	10.67	67	13.07	86
	secondary vocational	22.24	286	23.09	145	21.43	141
	secondary	38.34	493	39.17	246	37.54	247
	University	27.53	354	27.07	170	27.96	184
Net monthly household income	not more than 1000 PLN	5.37	69	5.10	32	5.62	37
	1001–1500 PLN	9.80	126	8.12	51	11.40	75
	1501–2000 PLN	11.12	143	11.46	72	10.79	71
	2001–3000 PLN	21.85	281	22.61	142	21.12	139
	3001–5000 PLN	10.50	135	10.51	66	10.49	69
	5001–7000 PLN	13.92	179	15.76	99	12.16	80
	more than 7000 PLN	5.52	71	4.94	31	6.08	40
	not revealed	21.93	282	21.50	135	22.34	147
Vocational status	employee	64.54	830	66.88	420	62.31	410
	self-employed or farmer	12.60	162	12.10	76	13.07	86
	retired or on disability pension	5.13	66	5.10	32	5.17	34
	high school or university student	8.79	113	7.64	48	9.88	65
	vocationally passive incl. unemployed	16.02	206	15.13	95	16.87	111
Marital status	single	28.46	366	27.87	175	29.03	191
	married	47.74	614	49.04	308	46.50	306
	in partnership	16.10	207	15.45	97	16.72	110
	widowed	2.18	28	2.23	14	2.13	14
	divorced or in separation	5.52	71	5.41	34	5.62	37

**Table 2:** Bivariate correlations of SFLQ items.

SFLQ Item	Item 1	Item 2	Item 3	Item 4	Item 5	Item 6	Item 7	Item 8	Item 9	Item 10	Item 11
item 2	0.47										
item 3	0.40	0.33									
item 4	0.33	0.32	0.62								
item 5	0.28	0.28	0.54	0.70							
item 6	0.45	0.31	0.36	0.35	0.34						
item 7	0.23	0.20	0.19	0.12	0.11	0.14					
item 8	0.53	0.53	0.47	0.42	0.39	0.44	0.31				
item 9	0.36	0.30	0.33	0.34	0.29	0.34	0.03	0.34			
item 10	0.32	0.28	0.36	0.35	0.32	0.36	0.08	0.37	0.67		
item 11	0.38	0.32	0.34	0.31	0.30	0.38	0.03	0.39	0.58	0.61	
item 12	0.37	0.29	0.34	0.36	0.32	0.37	−0.001	0.38	0.45	0.58	0.60

**Table 3:** Total variance explained by the three-factor latent structure of the scale.

Factor	Initial Eigenvalues			Sum of Squared Loading after Extraction			Sums of Squared Loading after Rotation		
	Total	% of Variance	Cumulated % of Variance	Total	% of Variance	Cumulated % of Variance	Total	% of Variance	Cumulated % of Variance
1	**4.99**	45.35	45.35	4.53	41.20	41.20	**2.35**	21.32	21.32
2	**1.35**	12.31	57.67	0.99	9.03	50.23	**1.94**	17.66	38.99
3	**1.07**	9.75	67.42	0.66	5.97	56.20	**1.89**	17.22	56.20
4	0.68	6.21	73.63						
5	0.57	5.16	78.79						
6	0.51	4.65	83.44						
7	0.45	4.06	87.50						
8	0.40	3.67	91.17						
9	0.38	3.42	94.59						
10	0.32	2.88	97.47						
11	0.28	2.53	100.00						

Eigenvalues of extract factor of at least 1.00 were bolded.

**Table 4:** Factor loadings extracted with the maximum likelihood method and rotated with the varimax method.

Item	Factor 1	Factor 2	Factor 3
item 1	0.652	0.168	0.245
item 2	0.610	0.168	0.179
item 3	0.350	0.604	0.223
item 4	0.223	0.818	0.200
item 5	0.190	0.761	0.180
item 6	0.439	0.250	0.302
item 8	0.682	0.284	0.231
item 9	0.205	0.172	0.718
item 10	0.173	0.190	0.792
item 11	0.286	0.141	0.704
item 12	0.276	0.213	0.589

**Table 5:** Correlations of food literacy score, health literacy score, and frequencies of the consumption of selected foods and nutritional habits.

	Information Accessing	Knowledge	Information Appraisal	Food Literacy Score
Descriptive statistics				
Mean (SD)	11.36 (4.02)	8.19 (2.79)	11.34 (2.40)	30.89 (7.66)
Median (IQR)	12.00 (4.40)	9.00 (4.00)	12.00 (2.00)	31.60 (8.80)
Range	0–18.00	2.00–13.00	4.00–16.00	6–47
Range of possible scores	0–19	2–13	4–16	6–48
Correlations				
HL score	0.54 **	0.22 **	0.42 **	0.46 **
Frequency of the consumption of food categories				
Fruit and vegetables	0.26 **	0.24 **	0.17 **	0.28 **
Meat	0.05	0.09 *	0.01	0.02
Fish	0.15 **	0.17 **	0.10 **	0.17 **
Industrial sugar products	−0.01	−0.04	0.03	−0.01
Wholemeal bread	0.22 **	0.20 **	0.14 **	0.23 **
Nutritional habits				
Omitting breakfast	−0.14 **	−0.07	−0.08 *	−0.12 *
Irregular meals	−0.13 **	−0.12 *	−0.09 *	−0.14 **
Late supper	−0.07	−0.13 **	−0.04	−0.10 *
Supper as the most caloric meal	−0.16 **	−0.11 *	−0.15 **	−0.20 **

**—*p*-value < 0.001, *—*p*-value from 0.001 to <0.05.

**Table 6:** The results of fitting the three-factor model.

Indexes	Threshold Levels of Indexes	Three-Factor Model (11 Items without Item 7)	One-Factor Model (11 Items without Item 7)
CDFR	<2.0 (*p* > 0.05)	3.154 (<0.001)	15.831 (<0.001)
CFI	Acceptable 0.90–0.95, good: 0.97	0.972	0.775
GFI	Acceptable: ≥0.90 to <0.95, good: ≥0.95	0.963	0.794
AGFI	Acceptable: ≥0.90 to <0.95, good: ≥0.95	0.940	0.692
NFI	Acceptable: ≥0.90 to <0.95, good: ≥0.95	0.959	0.765
RMSEA (90%CI)	Acceptable: <0.08 to 0.05, good: <0.05	0.059 (0.047–0.070)	0.154 (0.144–0.164)

CDFR—chi2-to-degrees-of-freedom ratio (*p*-value), CFI—Bentler’s comparative fit index, RMSEA (90%CI)—root-mean-square error approximation (90% confidence limit), GFI—goodness-of-fit index, AGFI—adjusted GFI, NFI—Bentler–Bonett normed fit index.

## Data Availability

The data are not publicly available, due to privacy and ethical restrictions. The authors did not include the information about the study provided to the participants that the public access to the data obtained during the survey may be considered. Access to the data will be granted on a case-by-case basis on a justified request after receiving consent from the Bioethical Committee at Jagiellonian University.

## Supplementary Materials

The following supporting information can be downloaded at: https://www.mdpi.com/article/10.3390/ijerph19159710/s1. Supplementary Material File S1: Table S1 Means, item-to-total correlations, and Cronbach’s alphas after removing specific items of the Polish version of the Short Food Literacy Questionnaire (Pl-SFLQ); Table S2. Original German and adapted-to-Polish versions of the items included in the Short Food Literacy Questionnaire.
