# Supplementary material for: Validation of the Short Food Literacy Questionnaire in the Representative Sample of Polish Internet Users
Source: Int J Environ Res Public Health. 2022 Aug 6;19(15):9710. doi: 10.3390/ijerph19159710 (PMC9367856; doi:10.3390/ijerph19159710)
Supplement: Supplementary file 1 [file ijerph-19-09710-s001.zip › ijerph-1848098-supplementary.pdf]

Table S1. Means, item-to-total correlations, and Cronbach's alphas after removing specific items of the Polish version of the Short Food Literacy Questionnaire (Pl-SFLQ).

Item	Mean after removing item	Variance of the scale after removing item	Item-factor correlation	Cronbach's alpha after removing item	Initial communalities
item 1	30.41	54.67	0.60	0.82	0.41
item 2	30.15	55.53	0.53	0.83	0.35
item 3	30.49	51.62	0.61	0.82	0.48
item 4	30.45	58.77	0.60	0.82	0.58
item 5	30.49	59.36	0.55	0.83	0.53
item 6	30.80	56.84	0.54	0.83	0.32
item 7	30.89	58.72	0.23	0.86	0.14
item 8	30.10	49.75	0.68	0.81	0.50
item 9	30.44	60.84	0.52	0.83	0.51
item 10	30.41	60.35	0.55	0.83	0.56
item 11	30.30	60.52	0.55	0.83	0.53
item 12	30.32	60.83	0.52	0.83	0.45

Table S2. Original German and adapted to Polish versions of the items included in the Short Food Literacy Questionnaire.

SFLQ item	German version	English version (for reference only)	Polish version
item 1	Wenn ich Fragen zu einer gesunden Ernährung habe, weiss ich wo ich mich informieren kann.	When I have questions on healthy nutrition, I know where I can find information on this issue.	Wiem, gdzie znaleźć odpowiednie informacje, gdy mam pytania dotyczące zdrowego odżywiania
item 2	Wie gut verstehen Sie im Allgemeinen folgende Informationen? Gemeint ist die Ver- ständlichkeit und nicht die Qualität der Information.<br>Bitte kreuzen Sie pro Zeile je eine Antwort an:	In general, how well do you understand the following types of nutritional information?	Ogólnie, jak dobrze rozumie Pan/Pani informacje dotyczące żywienia pochodzące z następujących źródeł. Chodzi o zrozumienie ich treści, a nie jej jakość. W przypadku każdego źródła, proszę zaznaczyć jedną odpowiedź.
item 3	Wie gut kennen Sie die Schweizer Lebensmittelpyramide?	How familiar are you with the Swiss Food Pyramid?	Jak dobrze zna Pan/Pani polskie zalecenia dotyczące zdrowego żywienia (Talerz Zdrowego Żywienia, Piramida Zdrowego Żywienia i Aktywności Fizycznej)?
item 4	Ich kenne die offiziellen Schweizer Empfehlungen zum Früchte- und Gemüseverzehr.	I know the official Swiss recommendations about fruit and vegetable consumption.	Znam oficjalne polskie zalecenia dotyczące spożycia owoców i warzyw.
item 5	Ich kenne die offizielle Schweizer Empfehlung zur Salzaufnahme.	I know the official Swiss recommendations about salt intake.	Znam oficjalne polskie zalecenia dotyczące spożycia soli.

item 6	An einem normalen Tag: Wie leicht oder schwer fällt es Ihnen zu Hause eine ausge- wogene Mahlzeit zusammenzustellen?	Think about a usual day: how easy or difficult is it for you to compose a balanced meal at home?	Jak łatwo lub trudno jest Panu/Pani przygotować w domu zbilansowany posiłek w zwykły dzień?
item 7	Und wie sieht es beim Thema Ernährung aus: Wie oft konnten Sie in der Vergangen- heit jemanden aus Ihrer Familie oder dem Bekanntenkreis bei Fragen zur Ernährung weiterhelfen?	In the past, how often were you able to help your family members or a friend if they had questions concerning nutritional issues?	Jak często w przeszłości, był Pan/Pani w stanie pomóc komuś z rodziny lub przyjaciół w kwestiach dotyczących odżywiania?
item 8	Es gibt heute viele Informationen zum Thema gesunde Ernährung. Wie gut gelingt es Ihnen die für Sie relevanten Informationen auszuwählen?	There is a lot of information available on healthy nutrition today. How well do you manage to choose the information relevant to you?	Obecnie dostępnych jest wiele informacji na temat zdrowego odżywiania. Jak dobrze radzi sobie Pan/Pani z wyborem informacji, które są dla Pana/Pani istotne?
item 9	Wie leicht fällt es Ihnen zu beurteilen, ob Informationen aus den Medien zum Thema Ernährung vertrauenswürdig sind?	How easy is it for you to judge if media information on nutritional issues can be trusted?	Jak łatwo jest Panu/Pani ocenić czy informacje dostępne w mediach na temat odżywiania są godne zaufania?
item 10	In der Werbung werden Lebensmittel häufig mit Gesundheit in Verbindung gebracht. Wie leicht fällt es Ihnen zu beurteilen, in wie fern die dargestellten Zusammenhänge zutreffen oder nicht?	Commercials often relate foods with health. How easy is it for you to judge if the presented associations are appropriate or not?	W reklamach produktów spożywczych często podkreśla się ich znaczenie dla zdrowia. Jak łatwo jest Panu/Pani ocenić czy te produkty mają rzeczywiście znaczenia dla zdrowia?
item 11	Wie leicht fällt es Ihnen einzuschätzen, ob ein Lebensmittel für eine gesunde Ernäh- rung von Bedeutung ist?	How easy is it for you to evaluate if a specific food is relevant for a healthy diet?	Jak łatwe jest dla Pana/Pani zdecydowanie czy dany rodzaj żywności ma istotne znaczenie dla zdrowego odżywiania?
item 12	Wie leicht fällt es Ihnen einzuschätzen, welchen Einfluss Ihre Essgewohnheiten lang- fristig auf Ihre Gesundheit haben können?	How easy is it for you to evaluate the longer-term impact of your dietary habits on your health?	Jak łatwe jest dla Pana/Pani określenie jaki wpływ Pana/Pani obecne zwyczaje żywieniowe będą miały na jego/jej zdrowie w przyszłości?
